# Veterinary Legislation and State Boards of Health

**Published:** 1899-04

**Authors:** 


### EDITORIAL.

### VETERINARY LEGISLATION AND STATE BOARDS OF HEALTH.

It is a noticeable fact that almost every legislator in the respective States is called upon to enact laws relating to veterinary and sanitary questions. These laws are year by year being more rigidly enforced. The boards are asking for more appropriations, which is just. And it is a surprising fact that in some States the Legislature is entirely ignorant of the amount of good that this State work is doing. If these State legislators were asked to appropriate the minimum sum for a State board of health to which the veterinary department is connected, and especially veterinary sanitation, many of them would invariably ridicule the veterinary service; but if these men were acquainted with the losses that occur through lack of such services, and with the good that the veterinarians can do their respective States, they would certainly vote for appropriation. For example, the State Legislature of California is now asked to enact some laws which if passed will be the best laws in the United States in regard to this particular subject; and these laws would probably never have been passed in that State had not the farmers and stock-raisers of the State been thoroughly frightened by the State becoming infected with Southern cattle-ticks which caused all the other States to quarantine against it. This has opened the eyes of the people, and to-day they are asking for protection. Minnesota has, we believe, the most conservative law in the Union in regard to this subject and one of the best men to enforce the State law. This can be clearly shown by the way that they have handled glanders, and by the way they have handled the quarantine system; and they have given other States something to go by. This question that is now being so vigorously taken up in Minnesota will, no doubt, be taken up in the future by other States; but, as we have intimated, it will take time for the legislators in other States to be taught the lesson that the State of Minnesota has already learned. There are many other States that probably would take this matter up, but they cannot secure the support of their legislators, and it is only hoped that Minnesota will give us more of an example in the future, and that she will not be cramped for funds, but can vigorously show us what can be done in the way of quarantine and the eradication of contagious diseases.

Every State veterinarian in other States of the Union should be part of the State board of health, and should work in his respective State to secure that kind of legislation that will enable him to be a part of the State board of health, and that will secure in the near future uniform laws in this direction. We only hope that it may be possible to secure laws in the direction of quarantine and investigation of imported stock, all to be uniform throughout the Union, and we believe that by the State boards having a veterinarian connected with them that it can very easily be perfected. We trust that the State of Nebraska, which is following up the laws of the State of Minnesota, will succeed in its law that is now before the Legislature, and that it will be as successful as that State in eradicating disease from its borders.

---

The Genessee Valley Veterinary Medical Association is to be commended for its strong and determined stand against "fake veterinary dentists." As a body these men are the worst parasites of the profession. Ignorant pretenders, the most of them, they have so little knowledge of what they profess to do that they invariably set up a more dangerous condition of the teeth than they remedy. Their methods of soliciting work are those of the vendor of quack nostrums, and their treatment of the truth unpardonable; their offensive egotism without a parallel and their whole conduct of the work they perform unprofessional, unscrupulous, and destructive of the good name and honor of the whole profession, and every veterinary organization of the land should take similar action against these wilful pretenders.

---

The Boston horseshow, commencing April 17th, will require all horses to be examined as to soundness and as to height before entering the show-ring. The following are listed as hereditary unsoundnesses applying to all breeding classes: Roaring, whistling, ringbone, spavin, navicular disease, and cataract.

